# The Changes of Leukocytes in Brain and Blood After Intracerebral Hemorrhage

**DOI:** 10.3389/fimmu.2021.617163

**Published:** 2021-02-15

**Authors:** Shuhao Mei, Yijie Shao, Yuanjian Fang, Jia'nan Lu, Jingwei Zheng, Shenbin Xu, Haijian Wu, Zeyu Sun, Jun Yu, Sheng Chen, Zhen Wang, Jianmin Zhang

**Affiliations:** ^1^Department of Neurosurgery, The Second Affiliated Hospital, Zhejiang University School of Medicine, Hangzhou, China; ^2^Brain Research Institute, Zhejiang University, Hangzhou, China; ^3^Collaborative Innovation Center for Brain Science, Zhejiang University, Hangzhou, China

**Keywords:** intracerebral hemorrhage, immune cells, infiltration, peripheral blood, natural killer cell, monocyte

## Abstract

Preclinical and clinical research has demonstrated that inflammation is a critical factor regulating intracerebral hemorrhage (ICH)-induced brain injury. Growing evidence suggests that myeloid cells and lymphocytes have an effect on the pathophysiological processes associated with ICH, such as inflammation, immune responses, perihematomal edema formation, blood–brain barrier (BBB) integrity, and cell death. However, the underlying mechanisms remain largely unknown. We aimed to explore the role immune cells played at different stages of the ICH. To achieve this, novel bioinformatics algorithms were employed to analyze the gene expression profiles and three different analytical tools were utilized to predict the abundances of cell types. In this study, we found that natural killer (NK) cells infiltrated into the brain parenchyma after ICH. Infiltrating NK cells may mediate brain injury through degranulation and recruitment of other cells. Besides, in the acute phase of ICH, monocytes in peripheral blood carried out phagocytosis and secretion of cytokines. On the other hand, in the subacute stage, non-classical monocytes were activated and showed a stronger ability to carry out heme metabolism, wound healing, and antigen processing and presentation. In conclusion, our findings emphasize the significance of intracerebral infiltrating immunocytes in ICH and demonstrate that ICH is a systemic disease affected by peripheral blood. The hub genes identified might be promising therapeutic targets. We also provide a reference on how to use bioinformatics approaches to explore non-neoplastic immune-related diseases.

## Introduction

Intracerebral hemorrhage (ICH) occurs when a cerebrovascular vessel ruptures, allowing blood flow into the brain parenchyma. It is the most common type of hemorrhagic stroke, accounting for about 15% of all strokes and resulting in approximately one in every three deaths within the first month of onset. Besides, the survivors show residual disability, a high risk of recurrent intracerebral hemorrhage, as well as other serious sequelae (1, 2). The high rate of mortality and morbidity associated with ICH can be partly attributed to the lack of curative treatments. Thus, a more comprehensive and in-depth understanding of this disease is urgently needed.

Previous studies have shown that inflammation precipitated by neuroglia and infiltrating leukocytes is a significant mechanism that participates in ICH-induced brain injury. Inflammation contributes to the breakdown of the blood-brain barrier (BBB), which in turn exacerbates the inflammation by allowing the infiltration of immunocytes (3–5). Besides, blood components, such as red blood cells (RBCs), leukocytes, and plasma proteins enter the brain immediately following the hemorrhage and these infiltrations may also play a role in the progress of the disease (6). Moreover, during the occurrence of ICH, there may be alterations in the cellular and molecular immune profiles of patients in the peripheral blood, which are highly correlated with prognosis and can therefore, act as prognostic biomarkers (7). There has been increased interest in the role of immune cells in ICH, but the exact underlying mechanism is still unknown. Different immune cells and molecules can either activate or suppress the immune system, and immune responses are a double-edged sword at different stages of the disease (8–11). Therefore, it is necessary to explore the dynamic changes in immune cells at different phases of ICH.

Over the past decade, the development of high-throughput technologies has made it possible to holistically analyze the alteration of transcriptome and proteome in different stages of diseases. Furthermore, the invention of novel algorithms including gene set enrichment analysis (GSEA) (12) and weighted correlation network analysis (WGCNA) (13), allows in depth analysis of variations in expression profiles. The establishment of databases, such as Gene Ontology (GO) (14, 15), and Kyoto Encyclopedia of Genes and Genomes (KEGG) (16, 17), advances our understanding of the associations between genes and biological processes.

In this study, we used bioinformatics to analyze data available in public databases to comprehensively explore the potent gene networks as well as immune cells that influence ICH. This study aimed to systematically portray the cellular and molecular immune changes in the brain and peripheral blood during the development of ICH. Elucidating these biological processes is beneficial to broaden our knowledge of ICH and might provide novel therapeutic targets for the disease.

## Materials and Methods

### Criteria for Data Selection and Data Collection

All articles containing ICH-related microarray or sequencing data were reviewed. Gene expression profiles were downloaded from the Gene Expression Omnibus (GEO) employing the R package GEOquery (18). The lists of differentially expressed genes (DEGs) were downloaded from Supplementary Material. The specific information of datasets and DEG lists that were included in this study are enumerated in Supplementary Table 1.

### Data Pre-processing

The expression matrixes downloaded from GEO were counter-checked to determine if there were any systematic errors or batch effects. The sva R package was used to remove batch effects (19). The RNA-sequencing (RNA-Seq) data were normalized using the trimmed mean of M-values (TMM) method (20).

### Construction of Co-expression Networks

The WGCNA R package (version 1.69) (13) was used to construct the co-expressed gene networks. Modules were generated using the dynamic tree-cutting function with the following parameters: TOMType = “signed,” minClusterSize = 100-3^*^deepSplit. Based on the recommendations that accompany the WGCNA package, we used Biweight midcorrelation instead of Pearson's correlation to calculate the correlations. Biweight midcorrelation allows the acquisition of more robust results. Gene significance (GS) is defined as the mediated *p*-value of each gene (GS = lgP) in the linear regression between gene expression and the clinical traits. Module eigengenes (MEs) are defined as the first principal components of each gene module, and the expression of MEs was considered as representative of all genes in a given module. kWithin, which means connectivity of each gene within a single module, was based on its *r*-values to all other genes within the same module. Correlation analysis was carried out between the MEs and the clinical traits in which a strong correlation was an indication of a close connection between the modules and the clinical traits.

### Gene Ontology and Pathway Analysis of Gene Expression Networks

Several modules were selected for further analysis based on their correlation with the clinical traits and the DEG lists. These modules were annotated using metascape (21).

### Gene Set Enrichment Analysis and Gene Set Variation Analysis (GSVA)

Gene set enrichment analysis and ridge plot were carried out using R package clusterProfiler (22). GSVA R package was utilized to evaluate the pathway enrichment for each sample (23). The annotated reference gene sets were downloaded from the molecular signatures database (MSigDB) (24). We further obtained the immune-related gene lists from ImmPort which provides accurate and timely scientific data in the area of immunological research (25).

### Estimation of Immune Cell Fractions

In order to ensure the robustness of the assessment results, we used three different methods that employ different principles. CIBERSORTx is an approach supporting deconvolution of microarray or bulk RNA-Seq data using signature genes and providing an estimate of the cell composition (26). xCell, a gene signatures-based method, allows the analysis of cellular heterogeneity based on tissue expression profiles (27). Immune Cell Abundance Identifier (ImmuCellAI) is introduced to precisely estimate the abundance of 24 immune cell types and the performance evaluation indicating that it is superiorly accurate on many T-cell subsets (28). The scale function in R was used to normalize the estimated proportions to allow comparison of the values obtained from the three methods. The consistency of results obtained from these three methods was evaluated using the intraclass correlation coefficient (ICC) utilizing irr R package (version 0.84.1) (29).

### Key Transcription Factors and Chromatin Regulators Discovery

Epigenetic Landscape *In Silico* deletion Analysis (LISA) was used to identify potential transcription factors (TFs) and chromatin regulators (CRs) that regulate the module genes and the DEGs. LISA is a powerful tool used to determine the TFs and the CRs that cause the perturbation of a differentially expressed gene set (30).

### Protein Expression Analysis

Data on protein expression levels were obtained from Genecards (31, 32), Proteomics DB (33), or The Human Protein Atlas (http://www.proteinatlas.org) (34).

### Receiver Operating Characteristic Curve

The receiver operating characteristic curve (ROC) and area under the curve (AUC) were calculated and plotted using the R package pROC (version 1.16.1) (35).

### Animals and Experimental ICH Surgery

Adult male C57BL/6 mice (20–25 g) purchased from Slac Laboratory Co., Ltd. (Shanghai, China) were used for this study. C57BL/6 mice were raised four per cage with controlled temperature and humidity, and in a 12-h light/dark cycle. All animal experimental protocols followed the Guide for the Care and Use of Laboratory Animals of the National Institutes of Health and were approved by the Institutional Animal Care and Use Committee of Zhejiang University. The ICH model was established as previously described (36). In brief, C57BL/6 mice were anesthetized by injecting pentobarbital (40 mg/kg) intraperitoneally. Under stereotactic guidance, a 1-mm-diameter burr hole was made in the skull (0.3 mm anterior to bregma and 2.5 mm right lateral to midline), then 30 μl of autologous blood from the femoral artery was injected into the right basal ganglia (3.5-mm depth below the skull) using a micro-perfusion pump at a rate of 3 μl/min. In case of the backflow of blood, the needle was gradually removed at 10 min after the completion of the injection.

### Immunofluorescence Staining

After anesthetized, the mice were euthanized. Transcardial perfusion was performed with 0.1 mmol phosphate-buffered saline (PBS) and 4% paraformaldehyde (PFA). The whole brain was immersed in 4% PFA for 8 h, then cryoprotected in serial 15 and 30% sucrose solutions. The brain samples were cut into coronal slices (8 μm) and fixed on slides. The sections were preprocessed with 10% donkey serum and 0.3% Triton X-100 and then incubated at 4°C with anti-CD45 antibody (1:200, Cell Signaling Technology Inc., Danvers, MA, USA, 55307S) and anti-ETS1 antibody (1:100, Cell Signaling Technology Inc., Danvers, MA, USA, 14069). After the incubation overnight, the cryosections were incubated with the secondary antibodies at 37°C for 1 h. Then, the fluorescence microscope (Olympus, Tokyo, Japan) was used to observe and capture the images.

### Statistical Analysis and Visualization

The student's *t*-test and the one-way ANOVA with *post-hoc* test were used to evaluate statistical differences. Results with *p* < 0.05 were regarded as statistically significant unless specified otherwise. Analyses were performed in R (version 3.6.3) and SPSS (version 22.0). Graphs were generated using ggplot2 (version 3.3.2), ggpubr (version 0.2.5), heatmap (version 1.0.12), and forestplot (version 1.10) from R.

## Results

### Functional Annotation and Proportion of Cells Inside the ICH Brain Samples

**Figure 1:**
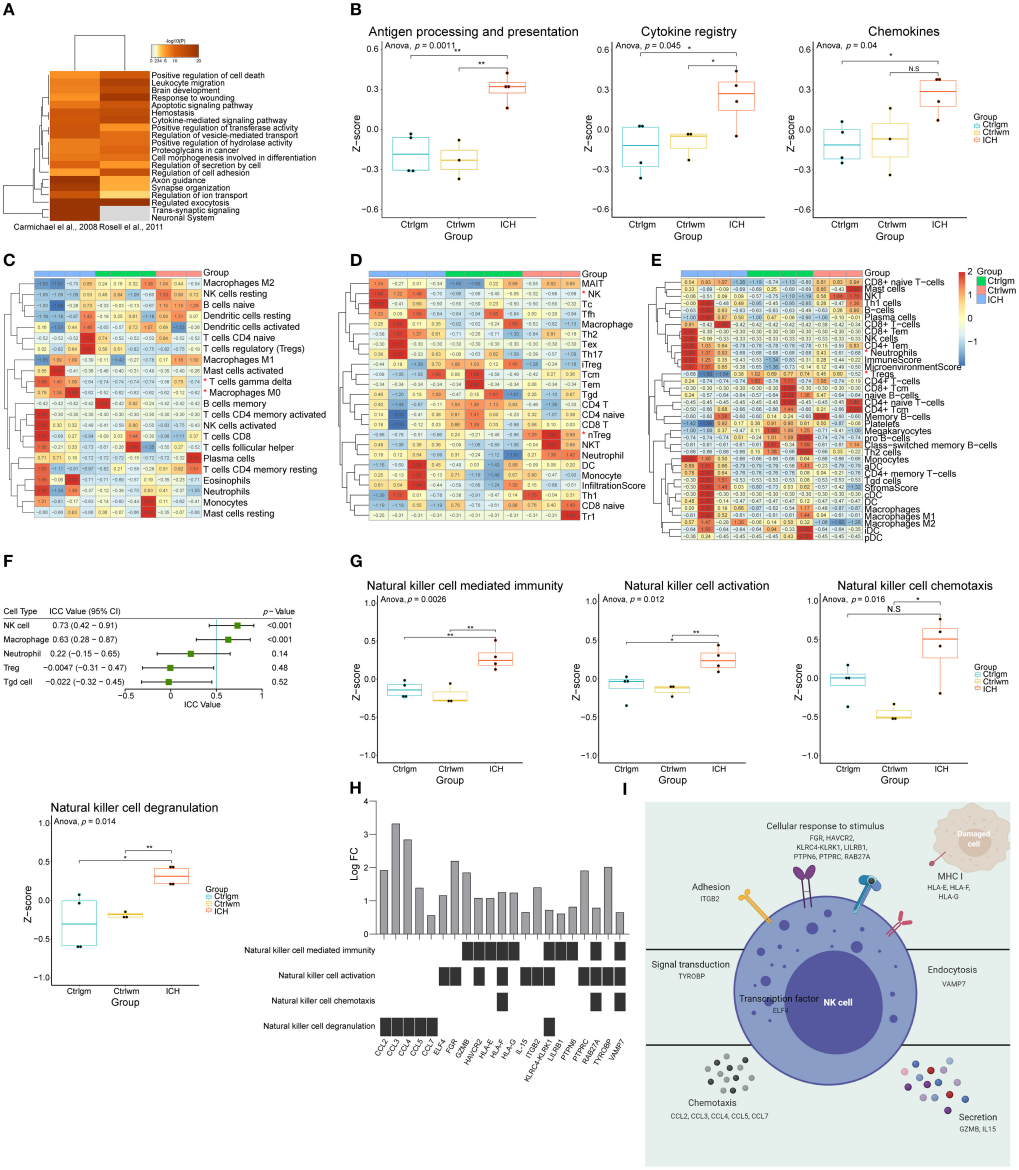
Function annotation and proportion of cells inside the ICH brain samples. **(A)** Gene functional enrichment of upregulated differentially expressed genes. **(B)** GSVA scores of differential immune-related pathways. **(C)** Heat map showed CIBERSORTx evaluation results of the immune cells proportion in each sample. **(D)** Heat map showed ImmuCellAI evaluation results of the immune cells proportion in each sample. **(E)** Heat map showed xCell evaluation results of the immune cells proportion in each sample. **(F)** Forest plot showed the ICC values of cell types, which indicated the consistency of the evaluation estimated by three different methods. **(G)** GSVA scores of NK cell-related biological processes. **(H)** The expression changes of DEGs correlated with NK cell function. **(I)** Schematic diagram of NK cell function. GSVA, gene set variation analysis; NK, natural killer; ICC, intraclass correlation coefficient; ICH, intracerebral hemorrhage; DEGs, differentially expressed genes.

First, to explore the main differences between the brain with ICH and control, the DEG lists from two independent studies were integrated for further analysis (37, 38). There were 584 upregulated genes and 508 downregulated genes in total. As shown in Figure 1A, overexpressed genes were significantly enriched in immune responses modulating leukocyte migration, adhesion, activation, and phagocytosis. Since the immune responses were the major biological process activated after ICH, we further performed GSVA on the expression matrices with reference to the molecular signatures of immune-related pathways downloaded from ImmPort. The GSVA scores of terms including antigen processing and presentation, cytokines, and chemokines were significantly higher in the ICH groups compared to the controls (Figure 1B). Immunocytes are principle components of immunity, thus, their proportions in each sample were calculated using three approaches. Results of CIBERSORTx analysis showed that the proportion of gamma delta T cells and macrophages were significantly higher in the ICH group (Figure 1C). On the other hand, results of ImmuCellAI analysis showed that natural killer (NK) cells were enriched and natural regulatory T (nTreg) cells were less (Figure 1D). In further contrast, findings from xCell analysis implied that neutrophils may infiltrate into the brain, and the regulatory T cells may decrease after ICH (Figure 1E). The outcomes obtained using the three methods were somewhat different, so we appraised the consistency with ICC. The three methods showed good uniformity in evaluating the proportion of NK cells (ICC value: 0.73, 95% confidence interval: 0.42–0.91, *p* < 0.001) and macrophages (ICC value: 0.63, 95% confidence interval: 0.28–0.87, *p* < 0.001, Figure 1F), indicating that the numbers for these two cells increase after damage. The three methods showed the best consistency in NK cell estimation, but its role in controlling brain damage is largely unknown. Consequently, we assessed the NK cells-related biological processes in each sample via GSVA. As shown in Figure 1G, the GSVA scores of NK-mediated immunity, cell activation, chemotaxis, and degranulation in perihematomal area samples were all higher than those in the contralateral areas. This suggests that the NK cells are recruited to the damaged site and become activated. Once activated, NK cells are able to undergo degranulation and perform cytotoxicity, by which they kill impaired cells, orchestrate other immune cells, and limit or aggravate immune responses (Figure 1I). Figure 1H displays the expression changes of differentially expressed genes which were correlated with NK cell function.

### Weighted Gene Co-expression Network Analysis

**Figure 2:**
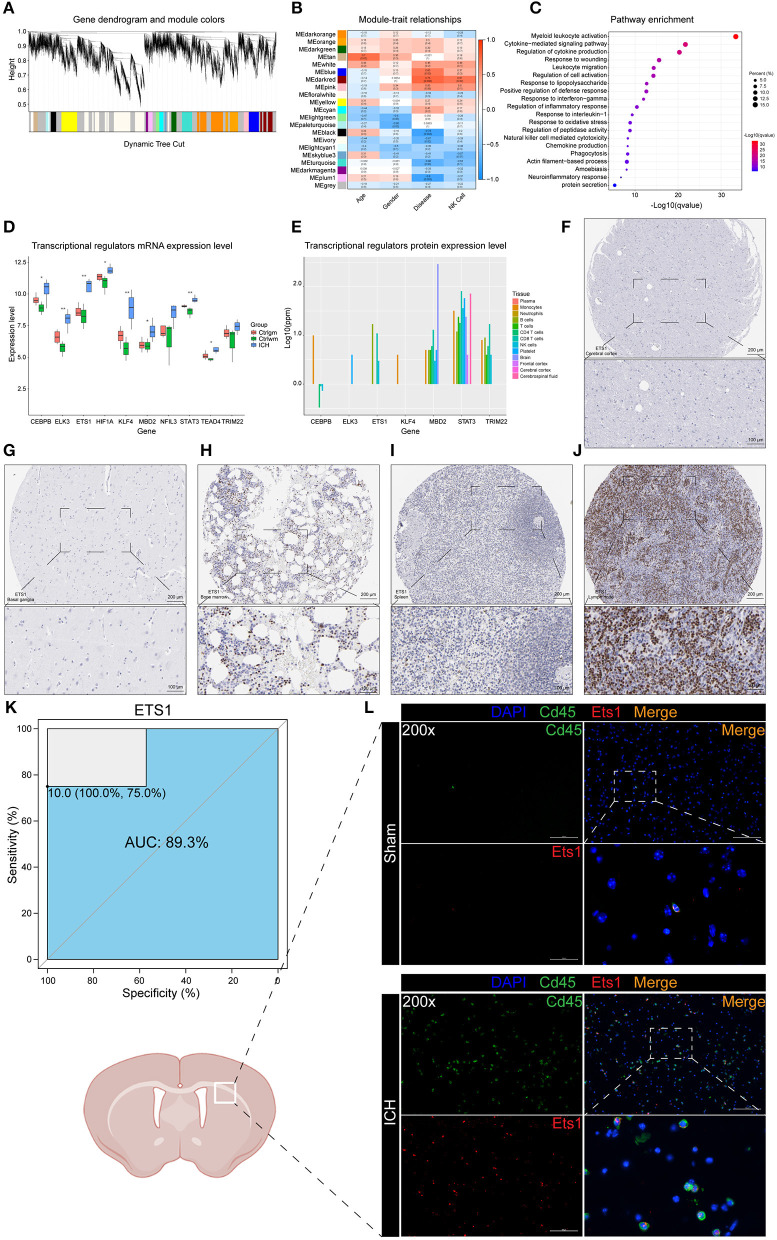
WGCNA and LISA identified key TRs that regulated ICH. **(A)** The cluster dendrogram constructing the gene modules. **(B)** Correlation analysis between the modules and the clinical traits. **(C)** Gene functional enrichment of darkred module. **(D)** mRNA expression level of TRs. **(E)** Protein expression level of TRs in different tissues of normal samples. **(F)** IHC result of ETS1 expression level in normal cerebral cortex. **(G)** IHC result of ETS1 expression level in normal basal ganglia. **(H)** IHC result of ETS1 expression level in normal bone marrow. **(I)** IHC result of ETS1 expression level in normal spleen. **(J)** IHC result of ETS1 expression level in normal lymph node. **(K)** ROC curve analysis of ETS1 in predicting ICH and the AUC value was 0.893. **(L)** Immunofluorescence staining results of ETS1 and CD45 in sham and ICH mice, *n* = 3. AUC, area under curve; IHC, immunohistochemistry; LISA, epigenetic landscape *in silico* deletion analysis; ROC, receiver operating characteristic; TRs, transcriptional regulators; WGCNA, weighted correlation network analysis; mRNA, Messenger RNA.

Weighted correlation network analysis has an advantage in analyzing samples with complex grouping as it is capable of screening for hub genes. In this study, the top 70% genes with the largest variance were inputted and β = 9 (no scale *R*^2^ = 0.89) was chosen to construct a scale-free network (Figure 2A, Supplementary Figure 1). To evaluate the module that is most closely associated with NK cells, the proportions of the NK cells in each sample were used as the phenotype trait. The highest correlation was observed between the darkred module and the cell fractions of the NK cells, implying that the darkred module might be a gene set associated with the biological function of NK cells (Figure 2B). Moreover, this module was also the most relevant module to ICH, which further proves that NK cells may be vital in directing ICH-induced brain injury. It is not surprising that the functional annotation of the darkred module showed that it is strongly correlated to immune response. Leukocyte activation, migration, phagocytosis, cytokine, and chemokine production were enriched (Figure 2C). To identify potential TFs and CRs that contribute to the perturbation of the darkred module, LISA was carried out and 1,316 transcriptional regulators (TRs) were ranked according to the *p*-value (Supplementary Table 2). We set stringent screening conditions to screen out the TRs that might be important in modulating the dark-red module, namely kWithin above median, *p*-value computed by LISA < 0.05, and belonging to the darkred module. Based on these conditions, 10 TRs were selected: ELK3, MBD2, KLF4, ETS1, STAT3, CEBPB, TEAD4, HIF1A, TRIM22, and NFIL3. All these TRs were highly expressed in the ICH samples (Figure 2D) indicating that their upregulation may be vital in regulating the pathophysiological process after ICH. To validate the above Messenger RNA (mRNA) results at the protein level, we detected the protein expression of these TRs in the immune system and central nervous system (CNS) via the GeneCards database. HIF1A was not recorded in the database and the protein expressions of NFIL3 and TEAD4 were not detected in the immune system and CNS. The remaining seven genes were all enriched in the immune cells (Figure 2E). According to the immunohistochemistry (IHC) results obtained from The Human Protein Atlas, ETS1 could not be detected in the normal brain parenchyma (including the cerebral cortex, the hippocampus, the basal ganglia, and the cerebellum). However, it was highly expressed in the bone marrow and the peripheral immune organs, such as the spleen and the lymph node (Figures 2F–J). The ROC curve of ETS1 revealed a good ability to distinguish between ICH and control specimens (AUC = 0.893; Figure 2K). To validate the upregulation of ETS1 and determine its localization, the mice suffering from ICH (24 h and 3 days post-ICH) were sacrificed for immunofluorescence staining (each group of three). In consistence with previous study (39), ETS1 can hardly be detected in the normal brain. It is no surprise that the number of CD45 positive cells in the brain parenchyma dramatically increased after damage. The ETS1 positive cells increased as well, and almost all of them were colocalized with CD45 (Figure 2L). These results imply that these TRs may regulate the infiltration of leukocytes and their activation might be significant in controlling ICH.

### Hub Genes Analysis

**Figure 3:**
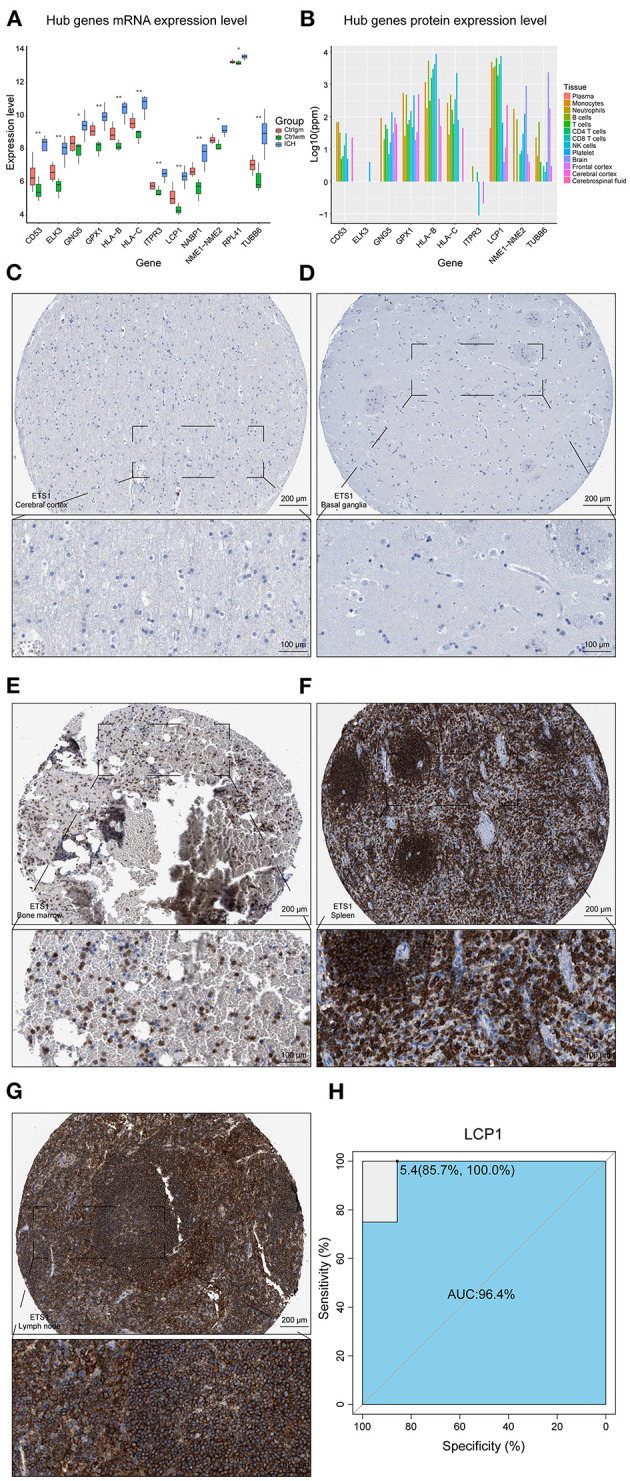
Hub gene analysis. **(A)** mRNA expression level of hub genes. **(B)** Protein expression level of hub genes in different tissues of normal samples. **(C)** IHC result of LCP1 expression level in normal cerebral cortex. **(D)** IHC result of LCP1 expression level in normal basal ganglia. **(E)** IHC result of LCP1 expression level in normal bone marrow. **(F)** IHC result of LCP1 expression level in normal spleen. **(G)** IHC result of LCP1 expression level in normal lymph node. **(H)** ROC curve analysis of LCP1 in predicting ICH and the AUC value was 0.964. AUC, area under curve; IHC, immunohistochemistry; ROC, receiver operating characteristic; mRNA, Messenger RNA.

The overlapping genes whose values were in the top 20 of kMEs and kWithin were selected out and regarded as darkred module hub genes. LCP1, HLA-C, HLA-B, ELK3, TUBB6, NME1-NME2, CD53, GPX1, RPL41, GNG5, ITPR3, and NABP1 were screened out and their mRNA expression levels were found to be high in ICH samples (Figure 3A). The upregulation of HLA-B, HLA-C, and LCP1 may indicate the infiltration of peripheral immune cells after ICH, since they were highly expressed in the immune cells (Figure 3B). Like ETS1, LCP1 was predominantly localized in the immune cells and was rarely detected in the normal brain parenchyma (Figures 3C–G). The ROC curve of LCP1 showed its promising ability to separate ICH from control samples (AUC = 0.964; Figure 3H). LCP1 had the strongest correlation between the darkred module and ICH as well as the highest kMEs and kWithin levels in this module. This was an indication that this gene might be critical in directing leukocyte infiltration, and infiltrating immune cells may play an important role in post-ICH pathophysiology.

### Peripheral Blood Changes in the Early Stage of ICH

**Figure 4:**
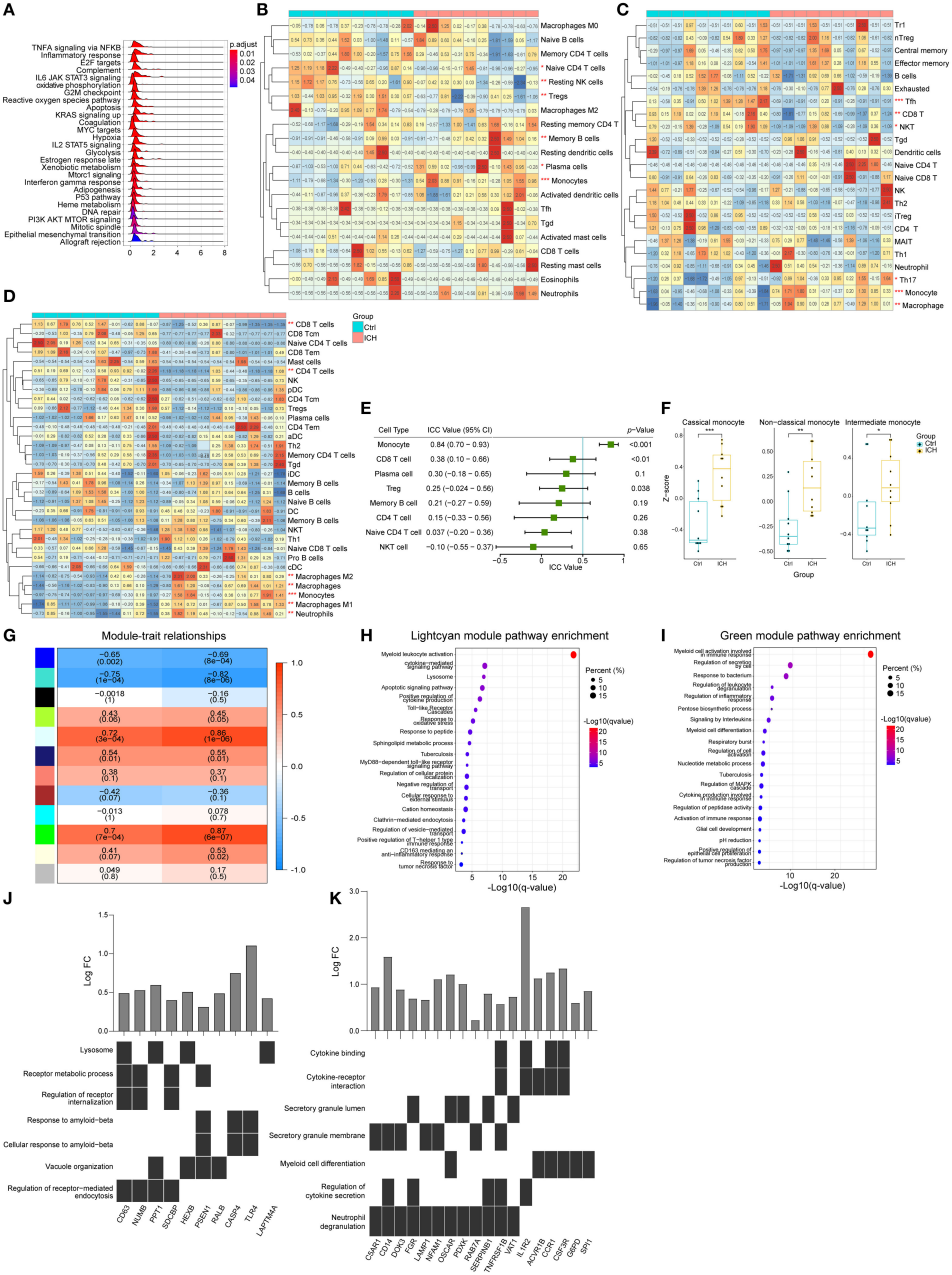
Peripheral blood changes in the early stage of ICH (6 h after onset). **(A)** Ridge plot showed GSEA result of GSE124624. **(B)** Heat map showed CIBERSORTx evaluation results of immune cells proportion in each sample. **(C)** Heat map showed ImmuCellAI evaluation results of immune cells proportion in each sample. **(D)** Heat map showed xCell evaluation results of immune cells proportion in each sample. **(E)** Forest plot showed the ICC values of cell types, which indicated the consistency of the evaluation estimated by the three different methods. **(F)** GSVA scores of marker genes of different subtypes monocytes. **(G)** Correlation analysis between the modules and clinical traits. **(H,I)** Gene functional enrichment of lightcyan and green module. **(J,K)** Functional annotation of hub genes belonging to lightcyan and green module. GSEA, gene set enrichment analysis GSVA, gene set variation analysis; ICC, intraclass correlation coefficient; ICH, intracerebral hemorrhage.

The aforementioned results suggested that the infiltration of peripheral immune cells may be vital in ICH-induced brain injury. Consequently, we used expression matrixes downloaded from GSE124624 and GSE125512 (40, 41) to explore the peripheral blood changes at different stages of the disease. First, we utilized the GSEA to evaluate the pathways that changed in peripheral leukocytes in the early stage (6 h after onset) of ICH. Like the brain specimen, the pathways that were perturbed were concentrated in immune responses, indicating that the immune system was activated 6 h after ICH (Figure 4A). We then estimated the cell proportions. CIBERSORTx analysis showed that the CD4 naïve T cells, resting NK cells, and regulatory T cells were significantly decreased, while memory B cells, plasma cells, and monocytes were increased (Figure 4B). In contrast, ImmuCellAI analysis showed that there was a decrease in follicular helper T cells (Tfh), CD8 T cells, and natural killer T (NKT) cells while there was an increase in T helper 17 (Th17) cells, monocytes, and macrophages (Figure 4C). On the other hand, the xCell analysis showed there was a decrease in CD8 T cells, CD4 T cells, CD4 naïve T cells, and Tregs, while there was an increase in the number of monocytes and macrophages (Figure 4D). Since the samples were mainly derived from peripheral blood mononuclear cells (PBMCs), granulocytes that do not belong to PBMCs and macrophages which were known as monocytes in blood were not discussed in the subsequent analysis. In summary, the proportion of T lymphocytes decreased while the monocytes increased. There were some dissimilarities among the three methods, thus, we evaluated the consistency of the results. In the ICC evaluation, the three methods showed the best consistency in monocyte estimation (ICC value: 0.84, 95% confidence interval: 0.70–0.93, *p* < 0.001, Figure 4E). Monocytes can be divided into three different subtypes: classical, non-classical, and intermediate (42, 43). Using marker genes acquired from the single-cell analysis of blood (44), we used GSVA to score the three subpopulations. The GSVA scores of all subtypes were higher in the ICH group compared to pre-ICH (Figure 4F). WGCNA was performed and two modules (lightcyan and green) showed a strong positive correlation with the monocyte and the disease (Figure 4G). Pathway enrichment analysis and functional annotation revealed that these two modules were correlated with myeloid leukocyte activation, cytokines secretion, inflammatory responses, lysosome formation, apoptosis, and oxidative stress, among others (Figures 4H,I). Hub genes in the two modules with the top 30 kMEs and kWithin values were selected out. The lightcyan hub genes were enriched in endocytosis and lysosome formation, which indicated that the phagocytic ability of monocytes increased after ICH (Figure 4J). Key genes in the green module were associated with cytokine secretion, binding, and cytokine-receptor interaction (Figure 4K), implying that monocytes participate in immune response via secreting cytokines.

### Differences of Peripheral Blood Between Acute and Subacute Stages of ICH

**Figure 5:**
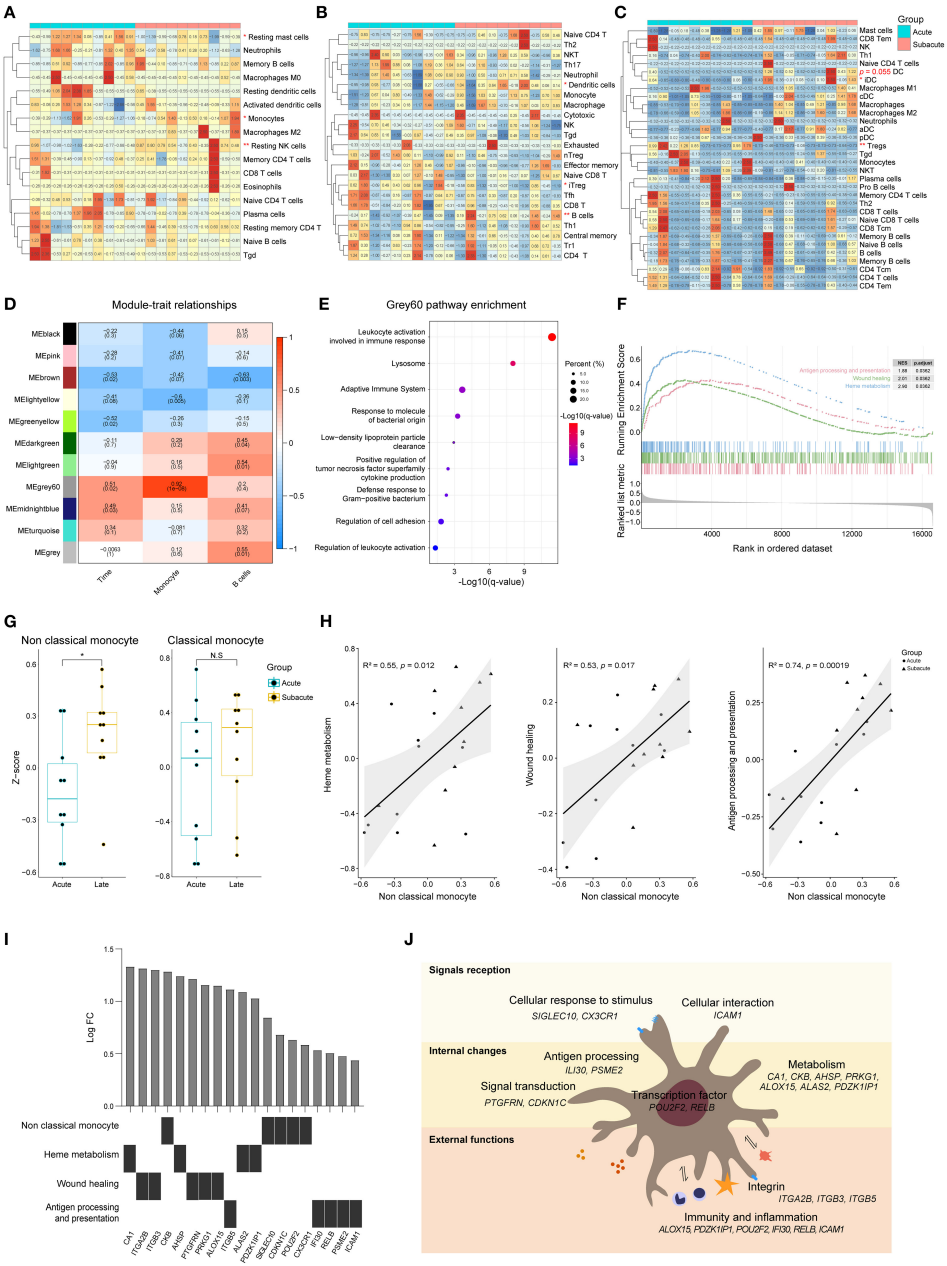
Differences of peripheral blood between acute and subacute stages of ICH. **(A)** Heat map showed CIBERSORTx evaluation results of immune cells proportion in each sample. **(B)** Heat map showed ImmuCellAI evaluation results of the immune cell proportion in each sample. **(C)** Heat map showed xCell evaluation results of the immune cell proportion in each sample. **(D)** Correlation analysis between the modules and clinical traits. **(E)** Gene functional enrichment of grey60 module. **(F)** GSEA for the antigen processing and presentation, wound healing and heme metabolism gene sets. **(G)** GSVA scores of marker genes of non-classical monocyte and classical monocyte. **(H)** Correlation of the GSVA scores of heme metabolism, wound healing and antigen processing and presentation with the GSVA score of non-classical monocyte. Biweight midcorrelation coefficient values are shown. **(I)** Top five differentially expressed genes related to heme metabolism, wound healing and antigen processing and presentation. **(J)** Schematic diagram of non-classical monocyte function. GSEA, gene set enrichment analysis; GSVA, gene set variation analysis; ICH, intracerebral hemorrhage.

CIBERSORTx analysis showed that there was a decrease in the proportion of resting mast cells as well as an increase in the proportion of resting NK cells and monocytes in the subacute phase samples (collected 72 ± 6 h following the acute ones), compared to the samples from the acute phase (within 24 h of ICH symptom onset) (Figure 5A). The results acquired from an ImmuCellAI analysis showed that there was a decrease in the induced Treg cells, while there was an increase in the B cells as well as dendritic cells (DCs) in the subacute phase samples (Figure 5B). Based on the xCell analysis, there was a decrease in the Treg cells and an increase in DCs (*p* = 0.055, Figure 5C). ICC analysis showed a moderate degree of consistency among the three methods in assessing the B cells. WGCNA analysis showed that there was no positive correlation between the modules and the B cells as well as the time of ICH onset. However, it was interesting that grey60, a module that was strongly associated with monocyte, was associated with the phase of the disease (Figure 5D). The functional annotation showed that this module may be involved in biological processes, such as immune response, lysosome formation, and adaptive immune system (Figure 5E).

Three biological processes, named heme metabolism, wound healing, and antigen processing and presentation, were enriched in the subacute phase (Figure 5F). To explore which subtype of monocyte is involved in biological changes at different phases of ICH, GSVA was performed. The GSVA score of non-classical monocyte was significantly higher in the subacute phase samples while there was no difference in the score of classical monocytes (Figure 5G). The results of correlation analysis revealed that the enhancement of heme metabolism, wound healing and antigen processing and presentation might be relevant to the non-classical monocyte (Figure 5H). Figure 5I shows the top five differentially expressed genes that were related to these three processes. Non-classical monocyte may participate in ICH via clearing hematoma, repairing damage, and presenting an antigen (Figure 5J).

## Discussion

In this study, the roles that peripheral immune cells played in the brain and blood of patients with ICH explored. The infiltration of leukocytes was assessed via the three different methods named CIBERSORTx, ImmuCellAI, and xCell. The activation of the immune system was one of the most prominent features in the patients with ICH when compared to the normal control tissue. In the perihematomal area, there was an increase in the NK cells, macrophages, and neutrophils but a decrease in Tregs. Activated NK cells mediate immunity through degranulation and recruitment of other cells. WGCNA analysis on brain tissue identified a module that was highly positively correlated with ICH immunological processes. Two hub genes in this module, ETS1 and LCP1, were significantly expressed in peripheral immune organs including the spleen, lymph node, and bone marrow. These genes are usually not detected in the normal brain, implying that the upregulation of ETS1 and LCP1 is mainly due to the infiltration of immunocytes. These two genes were also able to distinguish between ICH and control, which further indicated that peripheral immune cell infiltration played an extremely critical role in regulating the pathophysiological processes after ICH. During the early stages of ICH, monocyte activation was one of the most prominent characteristics in the peripheral blood. They showed stronger abilities to secrete cytokines and carry out endocytosis. In the subacute stage, heme metabolism, antigen processing and presentation, and wound healing were significantly enhanced, which might be associated with non-classical monocytes. The cell proportion estimation results implied the proliferation of B cells and dendritic cells in the subacute stage of the disease. The enrichment of antigen processing and presentation-related genes in this stage further indicated the presence of adaptive immunity. Moreover, enrichment of adaptive immune system-related genes in grey60 module suggested that monocyte might be a significant bridge between innate and adaptive immune in the subacute stage of ICH.

Preclinical and clinical research has shown that inflammation is a key factor regulating ICH-induced brain injury (6, 8, 45). Growing evidence suggests that myeloid cells and lymphocytes have an effect on immune responses, cell migration, perihematomal edema formation, BBB integrity, and cell death after ICH (46–49). However, the underlying mechanisms are largely unknown. A recent study revealed that NK cells could perform cytotoxicity and proinflammatory features, accelerating brain edema. Besides, brain-infiltrating NK cells can cooperate with neutrophils to break the BBB, which demonstrates that NK cells are capable of orchestrating immune response with other immunocytes (50). These findings are consistent with our results. A previous study showed that ETS1, one of the key TRs in the darkred module, can regulate the expression of various genes in NK cells involved in apoptosis and activation. In addition, ETS1 is a significant regulator of NK cell development as well as differentiation. NK cells lacking ETS1 fail to degranulate (51, 52). In patients with ICH, NK cells may facilitate brain edema, BBB destruction, and neuronal apoptosis via upregulation of ETS1. The presence of LCP1 in the darkred module as well as its unique expression in hematopoietic cell lineages emphasizes the significant role played by lymphocytes in ICH. A study report suggested that LCP1 may affect stroke-induced brain injury by influencing cell migration, phagocytosis, and pro-inflammatory activities (53). However, the specific role that LCP1 plays in ICH needs further exploration.

Chang and his colleagues have demonstrated that infiltrating monocyte-derived macrophages (MDMs) are essential to hematoma clearance and recovery after ICH. The activation of AXL/MERTK and erythrocyte phagocytosis is the underlying mechanism modulating this function (54). Our study further indicated that there might be a high positive correlation between peripheral blood monocytes and ICH. Hub-gene analysis indicated that the endocytosis of monocytes might be enhanced, and the lysosome was activated after ICH. These processes might be regulated by CD63, NUMB, PPT1, SDCBP, HEXB, and TLR4. Our study extends Chang's results, suggesting that phagocytosis and the recovery associated phenotype of MDMs may have been present in the monocyte phase. Besides AXL/MERTK, there might be many other pathways that were relevant to controlling this phenotype. Another module associated with ICH indicated that monocytes may influence brain injury via regulating the proinflammatory and anti-inflammatory dynamic balance of cytokines. The hub genes in the green module, CD14, CCR1, SPI1, and ACVR1B, were associated with the differentiation and maturation of monocytes (55–58). The upregulation of C5AR1, OSCAR, FGR, and NFAM1 may be proinflammatory and may promote cytokine and chemokine secretion. On the other hand, the activation of TNFRSF1B, SERPINB1, and IL1R2 might be anti-inflammatory (59–64). Interestingly, another two genes that are associated with metabolism, named G6PD and PDXK (65, 66), were also upregulated and separated into the green module after ICH. This result suggested that metabolic changes which is a distinctive feature after ICH (67), might modulate ICH via affecting the immune function of monocytes.

There may be an increase in non-classical monocytes that enhance heme metabolism, wound healing, and antigen presentation functions after ICH. The role of non-classical monocytes in ICH is largely unknown. Enhanced heme metabolism may promote the monocyte-mediated hematoma clearance. Some studies have demonstrated that this subtype was able to patrol the luminal surface of the endothelium, repair the damaged endothelium, and clear the cellular debris (68). Based on these results, we hypothesize that non-classical monocytes may be involved in the restoration of ICH-induced BBB damage. The ability of non-classical monocytes to facilitate CD4+ T-cell migration was also observed *in vitro* (69). Combined with improved antigen processing and presentation ability, non-classical monocytes might regulate the adaptive immunity after brain injury. The proliferation of B cells and DCs also suggested the emergence of adaptive immunity in the subacute stage of the disease. Previously, analytic tools which estimate the abundance of immune cell types are predominantly used to evaluate tumor-related samples. Our study suggested that these methods may also be powerful tools for exploring other immune-related diseases.

There are limitations to this study. First, the findings were largely based on transcriptome results, and transcriptome data can only reflect the perturbation of immune responses to a certain extent. Second, the reliability of our results was not determined using experiments. Consequently, further studies are required to check the dependability of our findings and explore the underlying mechanisms. Third, the number of cases adopted in this study was small. Thus, the robustness and generalizability of our findings need further verification in larger cohort studies.

## Conclusions

In summary, the significance of peripheral immune cells infiltration after ICH was emphasized. We identified leukocytes, including NK cells and monocytes, that might play an important role in brain injury induced by ICH. We also clarified the potential mechanisms as well as the key regulatory molecules. Besides infiltrating immunocytes, the dynamic changes of immune cells in the peripheral blood and their function in ICH were explored. We identified a few hub genes and potential pathways which might be essential to ICH-induced brain damage. Though with some limitations, this study provided a novel perspective of peripheral immune cells in ICH and a reference on how to use bioinformatic approaches to explore immunological non-tumor diseases.

## Data Availability Statement

Publicly available datasets were analyzed in this study. This data can be found at: https://www.ncbi.nlm.nih.gov/geo/query/acc.cgi?acc=GSE24265; https://www.ncbi.nlm.nih.gov/geo/query/acc.cgi?acc=GSE125512; https://www.ncbi.nlm.nih.gov/geo/query/acc.cgi?acc=GSE124624; http://www.proteinatlas.org; https://www.genecards.org; https://www.proteomicsdb.org/.

## Ethics Statement

The animal study was reviewed and approved by Institutional Animal Care and Use Committee of Zhejiang University.

## Author Contributions

JZha was the principal investigator. SM and YS collected, analyzed, and interpreted the data, wrote the paper, and prepared the original figures. YF reviewed the immunohistochemical pictures and revised the statistical methodology. JL, JZhe, SX, HW, and ZS helped with the study design and visualization. JY, SC, ZW, and JZha critically revised the texts and figures. All authors helped to design and conduct this study, read, and approved the final manuscript.

## Conflict of Interest

The authors declare that the research was conducted in the absence of any commercial or financial relationships that could be construed as a potential conflict of interest.
