# Supplementary material for: The Changes of Leukocytes in Brain and Blood After Intracerebral Hemorrhage
Source: Front Immunol. 2021 Feb 15;12:617163. doi: 10.3389/fimmu.2021.617163 (PMC7917117; doi:10.3389/fimmu.2021.617163)
Supplement: Supplementary file 1 [file Table_1.DOCX]

| GEO Accession | Source | Group | Sample  Size | Platform | Reference |
| --- | --- | --- | --- | --- | --- |
| GSE24265 | Human  Brain | ICH,  Control grey matter,  Control white matter | 4, 4, 3 | GPL570 | Rosell et al., 2011 |
| GSE124624 | Pig  PBMC | ICH,  Control | 10, 10 | GPL26005 | Walsh et al., 2019 |
| GSE125512 | Human  PBMC | Within 24 hours (early),  72 hours after the first (late) | 11, 11 | GPL15433 | Walsh et al., 2019 |

Abbreviations: PBMC, Peripheral Blood Mononuclear Cell
