# Supplementary figures and images for: The Changes of Leukocytes in Brain and Blood After Intracerebral Hemorrhage
Source: Front Immunol. 2021 Feb 15;12:617163. doi: 10.3389/fimmu.2021.617163 (PMC7917117; doi:10.3389/fimmu.2021.617163)

# Scale independence

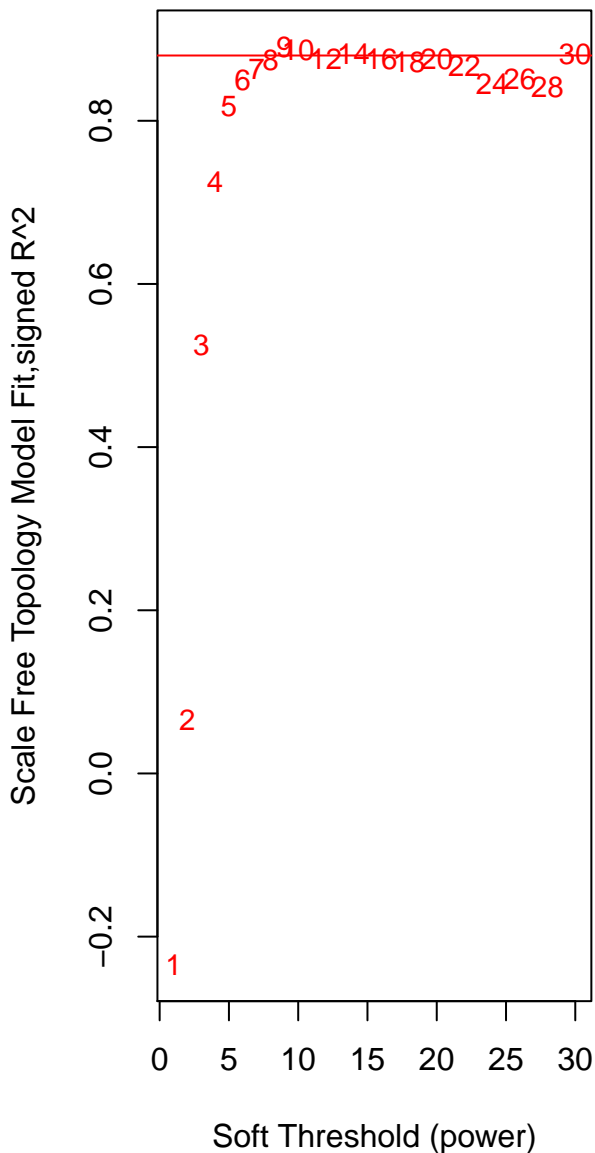

# Mean connectivity

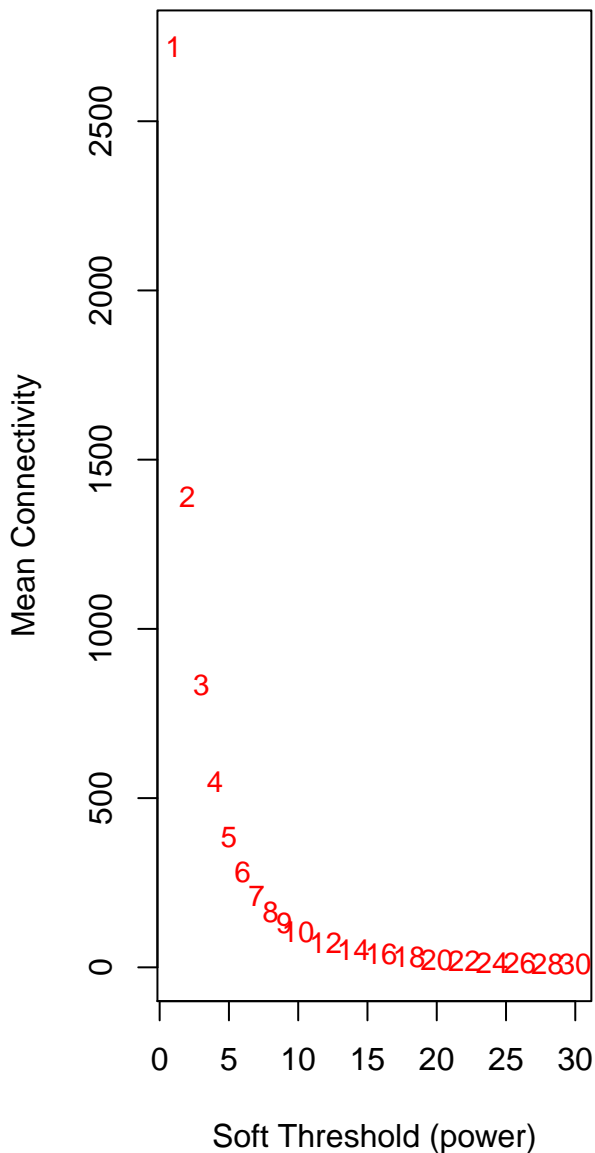

Supplement: Supplementary file 2 [file Data_Sheet_1.PDF]
